# *PagDET2* promotes cambium cell division and xylem differentiation in poplar stem

**DOI:** 10.3389/fpls.2022.923530

**Published:** 2022-08-26

**Authors:** Yao Wang, Yi Hao, Yakun Guo, Huixia Shou, Juan Du

**Affiliations:** ^1^State Key Laboratory of Plant Physiology and Biochemistry, College of Life Sciences, Zhejiang University, Hangzhou, China; ^2^State Key Laboratory of Subtropical Silviculture, School of Forestry and Biotechnology, Zhejiang Agriculture and Forestry University, Hangzhou, China

**Keywords:** biomass, castasterone, *DEETIOLATED2*, vascular cambium, xylem differentiation, poplar

## Abstract

Secondary growth of the woody tree stem is governed by meristematic cell division and differentiation in the vascular cambium. Multiple hormonal signals and endogenous developmental programs regulate vascular cambium activity. Brassinosteroids (BRs) significantly promote secondary stem growth and wood formation in poplar trees. However, the underlying regulatory mechanisms of BRs within the vascular tissue remain unclear. Genetic and anatomical approaches were used here to elucidate the role of *PagDET2*, the rate-limiting enzyme for BRs biosynthesis, in regulating secondary vascular cambium activity in *Populus*. This study showed that the elevated endogenous castasterone (CS) levels in tree stems through overexpressing *PagDET2* could enhance cambium meristem cell activity and xylem (XY) differentiation to promote secondary stem growth. RNA-seq analysis revealed that genes involved in BRs response, vascular cambium cell division, XY differentiation, and secondary cell wall synthesis were up-regulated.

## Introduction

Secondary meristem cells within the secondary growth woody tree stem undergo periclinal and anticlinal cell division and differentiation to generate secondary phloem (Ph) outward and secondary xylem (XY) (wood) inward during secondary growth within the woody stem (Larson and Isebrand, 1974). The secondary XY differentiation process includes XY mother cell division, cell expansion, secondary cell wall formation, and programmed cell death (Plomion et al., 2001).

Multiple hormones regulate the cambium meristem activities in poplar trees. Auxin is important for regulating the cambium cell division and differentiation (Sundberg et al., 2000; Nilsson et al., 2008). Ectopic expression of cytokinin oxidase gene *CKX* can reduce cytokinin content in the cambium zone (CZ) and inhibit the apical growth and radial growth of transgenic poplar (Kakimoto, 2003; Nieminen et al., 2008). Overexpression of *IPT7*, which encodes a cytokinin synthase in poplar, can promote cell division in the CZ and increase the contents of cytokinin and auxin (Tuominen et al., 1997; Immanen et al., 2016). Overexpression of the *GA20* oxidase gene in hybrid poplar can increase gibberellin content, promote XY differentiation, and increase the cell length of wood fibers (Eriksson et al., 2000; Mauriat and Moritz, 2009). Treatment of hybrid poplar with gaseous ethylene or ethylene precursor ACC can promote cell division and wood formation in poplar trees (Love et al., 2009; Etchells et al., 2012). Overexpression of *PttACO1* can promote XY differentiation (Andersson-Gunneras et al., 2003; Love et al., 2009).

Brassinosteroids (BRs) are plant-specific steroid hormones that can promote secondary XY differentiation in poplar trees (Jin et al., 2017; Shen et al., 2018; Du et al., 2020; Fan et al., 2020). More than 70 active BRs synthesized from campesterol have been identified in the plant Kingdome. There are significant differences in end-products of BRs biosynthesis for angiosperms. The most active types of BRs are castasterone (CS) and brassinolide (BL), catalyzed by members of the *CYP85A* family of cytochrome P450 monooxygenases. In Arabidopsis and tomato (*Lycopersicon esculentum*), CS and BL are major bioactive BRs, and BL is the more active form than CS (Bishop et al., 2006). In contrast, rice seems to use CS as a bioactive BR instead of producing BL (Yamamuro et al., 2000). BRs are synthesized by a series of rate-limiting enzymes through early and late C-6 oxidation pathways (Fujioka and Yokota, 2003). *DWF4 (DWARF4)* encodes 22α-hydroxylase, and overexpression of *PtoDWF4* promotes wood growth and XY formation (Shen et al., 2018). *PtCYP85A3*, the last rate-limiting enzyme in the BR biosynthesis pathway in aspen hybrid, encodes a C-6 oxidase. Furthermore, overexpression of *PtCYP85A3* can elevate the content of CS and promote the poplar tree growth and XY formation without affecting the composition of cellulose and lignin, and cell wall thickness (Jin et al., 2017).

*DET2 (DEETIOLATED2)* encodes a 5α-reductase, an early rate-limiting enzyme in the BRs synthesis pathway. *DET2* acts at the second step in BL biosynthesis from (24R)-24-methylcholest-4-En-3-one to (24R)-24-methyl-5α-cholestan-3-one (Li et al., 1996; Fujioka et al., 1997; Noguchi et al., 1999). Overexpression of *PtoDET2* in *Populus tomentosa* can increase endogenous BRs content and increase the wood biomass (Fan et al., 2020). Furthermore, overexpression of *PagDET2* in *Populus alba* × *P. glandulosa* can increase wood formation (Du et al., 2020). However, the molecular function of *PagDET2* in vascular cambium cell division and differentiation needs further investigation. The molecular mechanism of BR’s function in regulating vascular cambium cell activity remains to be further elucidated.

In this study, the endogenous CS levels were elevated by overexpressing the rate-limiting enzyme *PagDET2* gene within the poplar stem. *PagDET2* overexpression lines showed increased CS level and enhanced cambium cell division and differentiation activity associated with the increased wood biomass. We further explored the gene expression changes in the primary stem (PS), bark (BA), and secondary XY domain of the *PagDET2* overexpression transgenic stems using RNA-seq methods. The results indicated that cell division, XY differentiation, and secondary cell wall synthesis-related genes were significantly altered in the *PagDET2* overexpression transgenic lines. This study provides candidates of BR responsive genes which function in vascular cambium cell division and wood development in poplar trees.

## Materials and methods

### Plant materials and growth conditions

The *Populus alba* × *P. glandulosa* “84K” was used in all experiments. Trees were transferred to soil and grown at 22°C for 16/8 h (light/dark). Poplar plants were watered according to the evapotranspiration demands during different growth stages.

### Sequence alignment and phylogenetic analysis

The amino acid sequences of DET2 proteins of different species: *Arabidopsis thaliana (At)*, *Populus trichocarpa (Potri)*, *Gossypium hirsutum (Gh)*, *Glycine max (Gm)*, *Oryza sativa (Os)*, *Ipomoea nil (In)*, *Lycopersicon esculentum (Le)*, and *Populus alba* × *P. glandulosa (Pag)* used in our experiments were obtained from the following website.^1^ Multiple sequence alignments were performed with MegAlign software.

### Construction of the *PagDET2* vector and generation of transgenic poplar plants

The full-length coding sequence of the *PagDET2* gene was cloned from the cDNA of 84K poplar using sequence-specific primers and was cloned into the overexpression vector pCAMBIA1300 driven by 35S promoter and transformed into poplar 84K. Fifteen independent transgenic lines were obtained and identified through quantitative reverse transcription-polymerase chain reaction (qRT-PCR). Three lines (*PagDET2*-OE1, *PagDET2*-OE2, and *PagDET2*-OE3) were used for further analysis according to their overexpression levels. The artificial microRNA was designed by amiRNA designer website.^2^ The miRNA sequence was selected as UACAAUCCAAACUUCCCUUU, and the corresponding target sequence was UUGUUAGUUGAAGGGAA. The knockdown expression vector driven by the 35S promoter was constructed. Ten independent transgenic lines were obtained and identified through qRT-PCR. Three lines (amiR-1, amiR-2, and amiR-3) were used for further analysis according to their expression levels. All primers used are listed in Supplementary Table 2. The transgenic materials used in this experiment were retransformed, completely different from the transgenic lines used in a previous study (Du et al., 2020).

### Brassinosteroid measurement

Brassinosteroids contents were detected by MetWare^3^ based on the AB Sciex QTRAP 6500 LC-MS/MS platform.

### Anatomical observations

Stems from wild-type and transgenic plants were fixed with 2.5% glutaraldehyde solution and subsequently passed over a graded ethanol series for histological observations. Then, the sections were embedded in resin. Sections (1–3 μm) were stained with 0.05% toluidine blue O (TBO) for 60 s and washed three times in water. Images were captured under a bright field using a LEICA DM6B microscope.

### Scanning electron microscopy

Cross-sections were obtained by dissecting transversely with a razor blade by hand, and the samples were fixed with 2.5% glutaraldehyde solution and subsequently passed over a graded ethanol series. The samples were dried in Hitachi HCP-2 critical point dryer. Then the samples were attached using double-sided sticky tapes and observed by the Hitachi SU-8010 scanning electron microscope (SEM).

### Statistical analysis

At least 3 biological repeats per transgenic line were analyzed using SPSS statistical software. Student’s *t*-test, *P < 0.05, **P < 0.01, ***P < 0.001 for statistical analysis.

### RNA-seq

The shoot apical meristem and the first, the second, and the third elongating stem internodes were collected and pooled together as the PS. The secondary stem internodes from 5th to 12th were cut. The BA tissues, including differentiating Ph and cambium meristem cells, were harvested. The surface tissues of the trunk were harvested as XY. The PS, BA, and XY from tree stems were sampled from the 84K wild-type control and *PagDET2* overexpression lines. Sample methods are shown in Supplementary Figure 5. Total RNA was extracted by RNeasy Micro Kit (Cat # 74004, Qiagen) according to the standard procedure. The qualified RNA could be used for subsequent sequencing experiments. Sequencing libraries were synthesized using the TruSeq^®^ RNA sample preparation kit (Illumina, San Diego, CA, United States). The concentration and size of the constructed library were detected using Qubit^®^ 2.0 Fluorometer and Agilent 2100, respectively. Double-end sequencing was performed using a paired-end program. The sequencing was controlled by the data collection software provided by Illumina, and real-time data analysis was performed. Library construction and sequencing were performed in Shanghai Biotechnology Corporation.

### Sequencing, data processing, and analysis

The cDNA was sequenced using a high-throughput sequencing instrument (Illumina HiSeq 2000/2500, MiSeq). The spliced mapping algorithm of Hisat2 (version: 2.0.4) is applied to genome mapping of pre-processed Readfield (Kim et al., 2015). The genome version is P. trichocarpa_444_v3.1. StringTie (version: 1.3.0) was used to count the number of Fragments of each gene after the Hisat2 comparison, and then the trimmed mean of M values (TMM) method was used to normalize the field (Mortazavi et al., 2008; Pertea et al., 2015). Finally, the FPKM value of each gene was calculated by Perl script. The edgeR was used to analyze the difference gene between samples, and the *P*-value was obtained and corrected by multiple hypothesis tests (Robinson et al., 2010). The differentially expressed genes (DEGs) were selected using the following filter criteria: FDR ≤ 0.05 and fold-change ≥ 2. The Gene Ontology (GO) enrichment analysis using A_*GRI*_GO_*V*_2.0 was performed using R scripts based on a hypergeometric test to examine the biological significance of the DEGs. The same principle as GO enrichment can also be used for KEGG pathway enrichment analysis of differential genes.

### Weighted gene co-expression network analysis

The correlation coefficient and the average connection degree of the connection degrees k and p(k) under each power value were calculated by setting a series of soft-thresholding power values. The cut Height = 0.99 and the gene number ≥ 50 within a module were used as the threshold. The similarity among the modules was calculated with the module eigengene, which was defined as the first principal component of a given module and could be considered a representative of the gene expression profiles in a module. Then the expression trends among PS, BA, and XY of each module were obtained using the expression values of genes.

## Results

### Phylogenetic and expression pattern analysis of *PagDET2*

**FIGURE 1:**
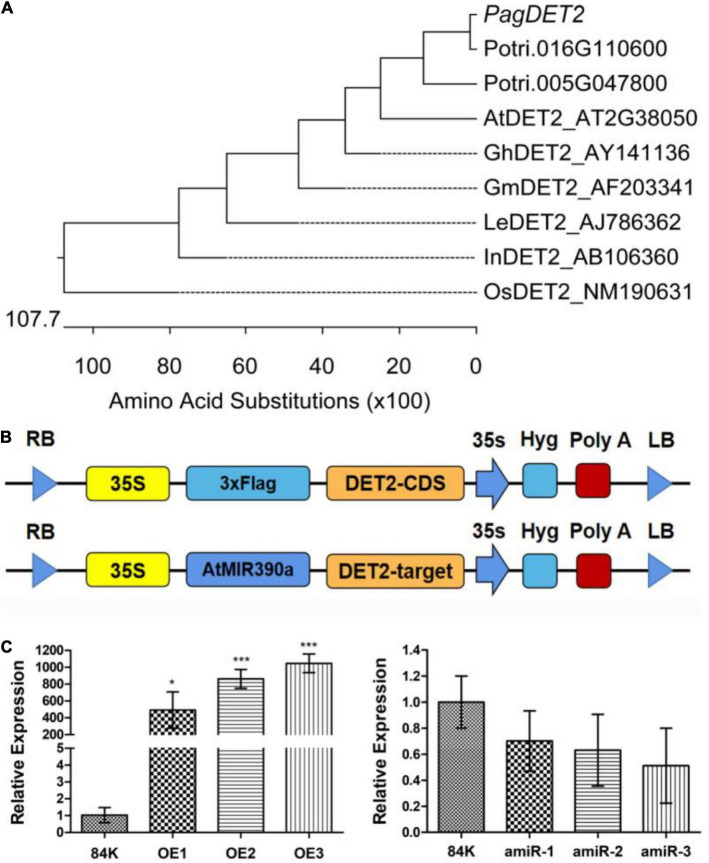
Phylogenetic analysis and identification of *PagDET2* transgenic lines. **(A)** The phylogenetic tree of *PagDET2* in different plant species was plotted by MegAlign software. **(B)** The schematic map of constitutive overexpression vector and artificial microRNA inhibition expression vector of *PagDET2*. **(C)** The transcription level of the *PagDET2* gene in different lines. Mean ± SD. * and *** indicated significant difference at *P* < 0.05 and *P* < 0.001, respectively (Student’s *t*-test).

The phylogenetic tree of *PagDET2* among different species was plotted according to the distance of genetic relationship (Figure 1A and Supplementary Table 1). Multiple alignments of amino acid sequences between different species are shown in Supplementary Figure 1. Two genes of *Populus trichocarpa* have higher homology with *AtDET2*, i.e., Potri.005G047800 and Potri.016G110600. The expression profiles of these two genes were found in AspWood (Sundell et al., 2017). Potri.016G110600 showed higher expression in the early development of the XY (Supplementary Figure 2), so its 84K homology was selected as the follow-up study.

### Overexpression of *PagDET2* elevated the content of castasterone and promoted the wood biomass in the poplar stem

Fifteen independent *PagDET2* overexpression lines and ten independent *PagDET2* knockdown lines were identified. The expression of *PagDET2* was detected by quantitative real-time PCR (Supplementary Table 2). Three independent transgenic lines that showed *PagDET2* up-regulation by 500-fold or greater based on qRT-PCR were selected for further study (Figures 1B,C).

**FIGURE 2:**
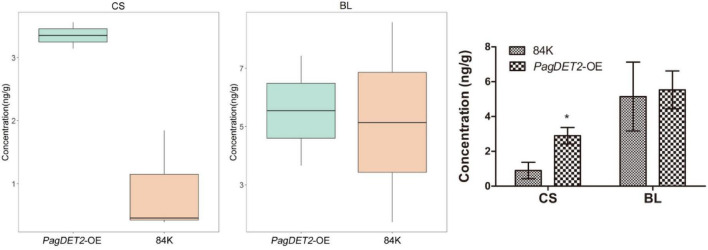
The CS and BL contents of *PagDET2* overexpression lines and wild-type controls. CS, castasterone; BL, brassinolide. Mean ± SD. *Indicate significant difference at *P* < 0.05 (Student’s *t*-test).

Brassinosteroids concentration was measured by Agilent 1290 Infinity LC ultra-performance liquid chromatography. Compared with the wild-type 84K, the concentration of CS was significantly elevated in *PagDET2*-OE lines, while that of BL did not show significant changes (Figure 2).

**FIGURE 3:**
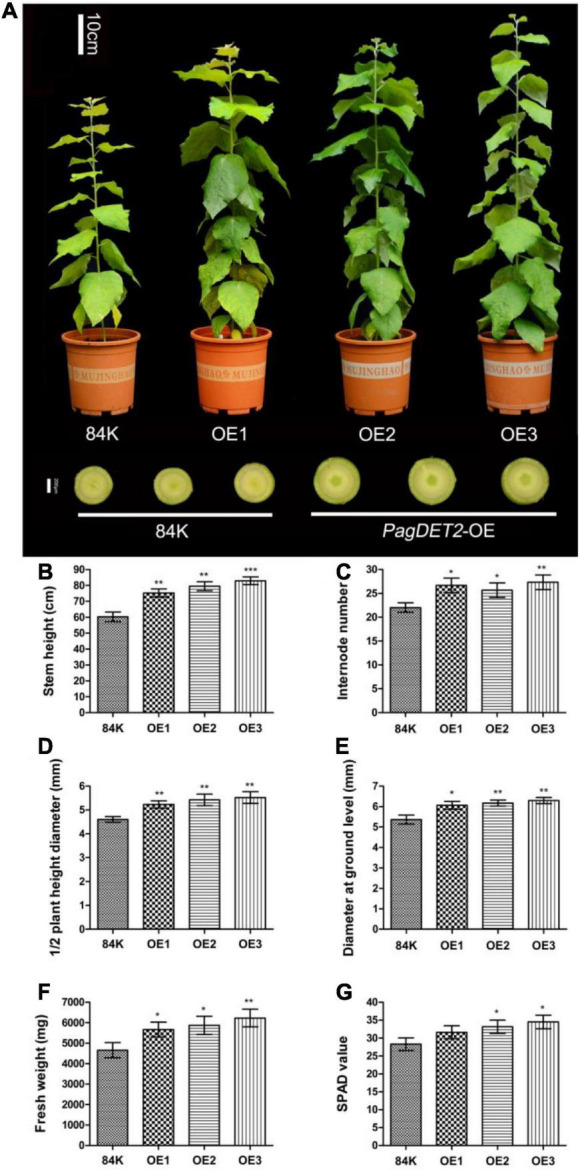
Growth comparisons of wild-type and *PagDET2* overexpression lines. **(A)** Phenotypes of 90-day-old 84K wild-type and *PagDET2* overexpression lines. **(B–G)** The stem height, internode number, 1/2 plant height diameter, ground diameter, fresh weight, and SPAD value of 84K wild-type and *PagDET2* overexpression lines. Mean ± SD. *, **, and *** showed significant difference at *P* < 0.05, *P* < 0.01, and *P* < 0.001, respectively (Student’s *t*-test).

The phenotype of *PagDET2* overexpression lines was positively correlated with the gene expression level, and all regenerated transgenic lines showed a similar phenotype with enhanced stem secondary growth and wood biomass accumulation (Figure 3A). The *PagDET2* transgenic lines used in this study showed higher expression levels than those in a previous study (Du et al., 2020). Due to the similarity of *PagDET2* knockdown lines and wild-type control 84K in stem phenotypes, they were not used for further research (Supplementary Figure 3).

*PagDET2*-OE lines exhibited a larger leaf area with dark green leaf color and a greater SPAD value than the wild-type controls. The stem phenotypes were characterized by elevated stem height and increased stem internode numbers and stem diameters (Figures 3A–E,G and Supplementary Table 3). Wood biomass was also significantly increased in the *PagDET2*-OE stems (Figure 3F and Supplementary Table 3), indicating that elevated CS levels in the *PagDET2*-OE lines could promote the wood biomass accumulation in poplar stem during secondary growth.

### *PagDET2* promotes the vascular cambium cells’ division and differentiation in poplar

**FIGURE 4:**
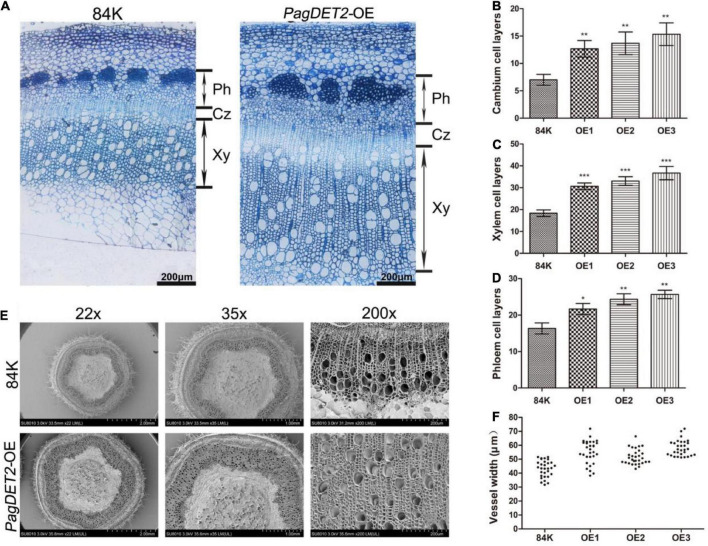
The 60-day-old wild-type and *PagDET2* overexpression lines anatomical observations and phenotypic statistics. **(A)** Toluidine blue stained cross-sections of the stem of 84K wild-type and *PagDET2* overexpression lines, bar = 100 μm. **(B–D)** The number of cambium cell layers, XY cell layers, and Ph cell layers of 84K wild-type and *PagDET2* overexpression lines. **(E)** SEM observation of 84K wild-type and *PagDET2* overexpression lines at different magnifications. **(F)** The vessel width of 84K wild-type and *PagDET2* overexpression lines was counted under SEM, and the width of 30 vessels was counted. CZ, cambium zone; XY, xylem; Ph, phloem; SEM, scanning electron microscope. Mean ± SD. *, **, and *** showed significant difference at *P* < 0.05, *P* < 0.01, and *P* < 0.001, respectively (Student’s *t*-test).

Histology analysis was performed for the vascular tissues of *PagDET2*-OE stems and controls to further study the regulation of elevated CS on cell division and differentiation in the secondary vascular tissues. The *PagDET2*-OE lines had changes in stem development along the stem internode IN 3, 5, 7, and 9 (Supplementary Figures 4A,B). During the early initiation stage of secondary growth, the 3rd internode of non-transgenic controls and *PagDET2*-OE lines showed a typical primary vascular tissue structure with a discontinuous XY ring and few enlarged XY vessels (Supplementary Figure 4A IN3). At the 5th internode, *PagDET2*-OE lines already had continuous secondary XY rings, which were lacking in wild-type controls (Supplementary Figure 4A IN5). Histology results showed that the 7th and 9th internode of *PagDET2*-OE lines exhibited increased secondary XY and Ph domains compared with matched wild-type controls (Supplementary Figure 4A IN7 and IN9). The statistical analysis of cell numbers in both Ph and XY domains was significantly increased in the *PagDET2*-OE lines compared with the wild-type controls (Supplementary Figures 4C,D). In addition, the width of the secondary XY vessels in *PagDET2*-OE lines was significantly larger than that of wild-type controls (Figures 4E,F and Supplementary Table 3).

The stem anatomy results of *PagDET2*-OE lines revealed that *PagDET2* overexpression could affect the timing of the development of secondary meristems and enhance the cell division rates of secondary meristems, thus further promoting the meristem differentiation toward XY and Ph direction, respectively (Figures 4A–D and Supplementary Table 3).

### *PagDET2* enhances the expression of cambium division and differentiation genes

Genome-wide transcriptome analysis was performed using the RNA-seq method to further determine biological processes linked to *PagDET2* action. Three trees of *PagDET2*-OE lines and wild-type controls were used for analysis. The RNA libraries were sampled from PS, BA, and XY of *PagDET2*-OE lines and wild-type controls (Supplementary Figure 5). RNA sequencing raw data can be obtained in the GEO repository. The differential genes of wild-type controls and *PagDET2*-OE lines were displayed according to tissue types.

**FIGURE 5:**
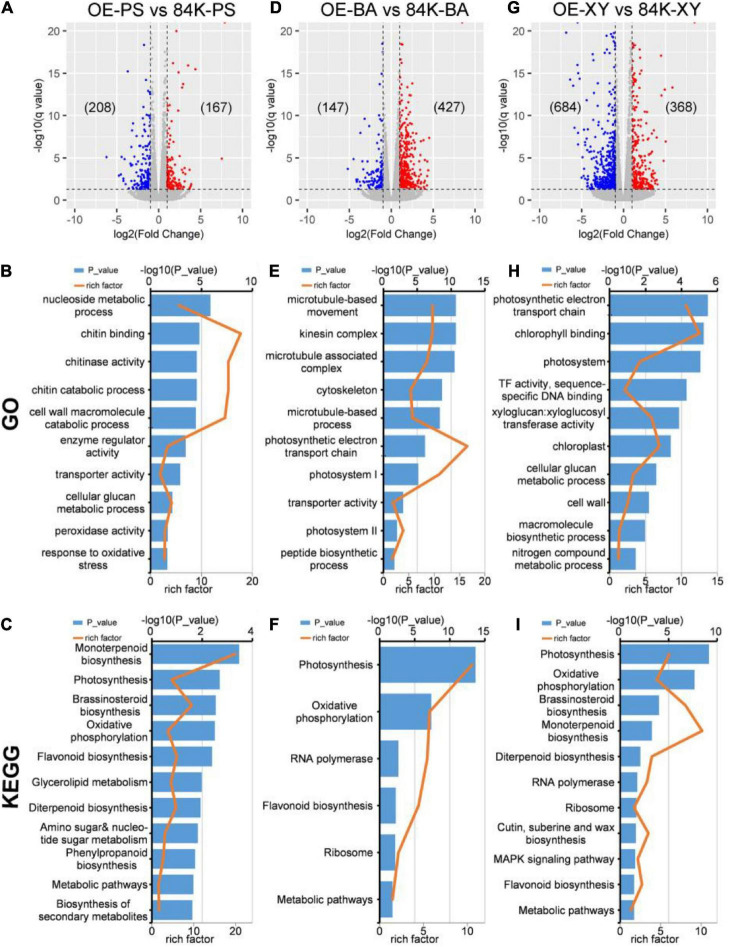
DEGs analysis of wild-type and *PagDET2* overexpression lines. **(A–C)** The volcano plot, GO enrichment analysis, KEGG enrichment analysis of PS domain; **(D–F)** the volcano plot, GO enrichment analysis, KEGG enrichment analysis of BA domain; **(G–I)** the volcano plot, GO enrichment analysis, KEGG enrichment analysis of XY domain. DEGs, differentially expressed genes; PS, primary stem; BA, bark; XY, xylem.

*PagDET2*-OE lines showed differential expression of 375 genes in PS, 574 genes in BA, and 1,052 genes in XY compared with wild-type controls (Figures 5A,D,G and Supplementary Tables 4–6). The DEGs of PS were associated with the biological processes related to photosynthesis, BRs biosynthesis, biosynthesis of secondary metabolites, cell wall macromolecule catabolic process, cellular glucan metabolic process, and oxidative stress (Figures 5B,C and Supplementary Tables 7, 10) according to GO and KEGG enrichment analyses. The DEGs of BA were associated with the peptide biosynthetic process, microtubule-based process, photosynthesis, and metabolic pathways (Figures 5E,F and Supplementary Tables 8, 11). The DEGs of XY were associated with cellular glucan metabolic process, macromolecule biosynthetic process, BRs biosynthesis, MAPK signaling pathway, photosynthesis, and nitrogen compound metabolic process (Figures 5H,I and Supplementary Tables 9, 12) according to GO and KEGG analyses.

**FIGURE 6:**
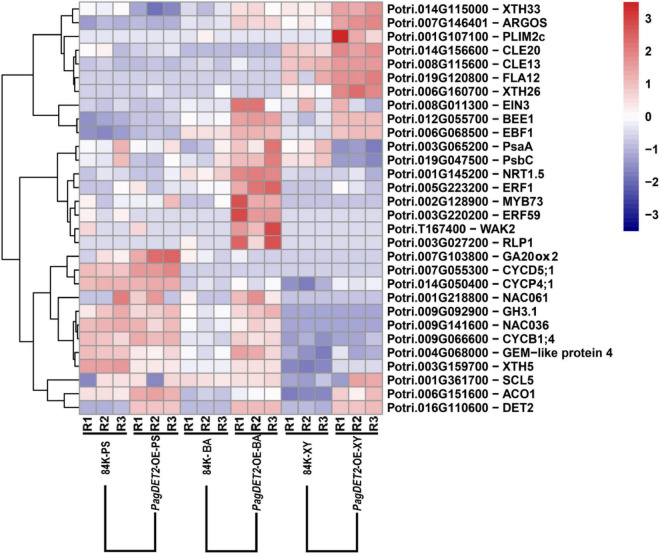
Representative gene expression in the heatmap. Heatmap shows representative gene expression across all the 18 samples, including cell cycle protein-encoding genes *CYCD*, *CYCB*; cell wall biosynthesis-related genes *XTHs*; transcription factor encoding gene *ERF1* and ethylene synthase encoding gene *ACO1* responding to ethylene signal.

**FIGURE 7:**
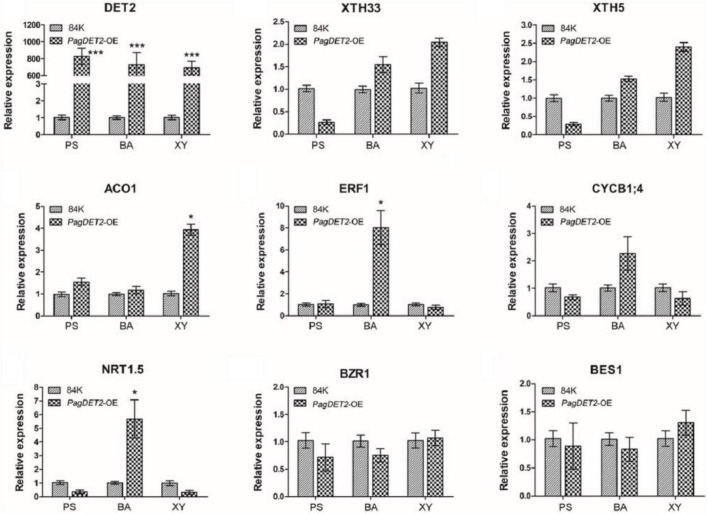
Expression analysis of representative genes in the PS, BA, and XY domains of 84K wild-type and *PagDET2* overexpression lines. PS, primary stem; BA, bark; XY, xylem. Mean ± SD. * and *** indicated significant difference at *P* < 0.05 and *P* < 0.001, respectively (Student’s *t*-test).

Representative DEGs are shown in Figure 6. Cambium cell division genes, including different genes for encoding cyclin family proteins (*CYCD5; 1*, *CYCP4; 1*, and *CYCB1; 4*), were significantly up-regulated in the PS and BA domain of *PagDET2*-OE lines (Supplementary Tables 4, 5). Secondary cell wall deposition-related genes, including *XTH5*, *XTH26*, and *XTH33*, were significantly up-regulated in the XY domain of *PagDET2*-OE lines (Supplementary Table 6). Many hormone-related genes had changes in transcription levels. Gibberellins synthesis-related gene *GA20ox2* was up-regulated in the PS domain (Supplementary Table 4). Ethylene synthase encoding gene *ACO1* was up-regulated in the XY domain, and ethylene signal transcription factor coding gene *ERF1* was up-regulated in the BA domain (Supplementary Tables 5, 6). Some of these genes were further verified by qRT-PCR (Figure 7).

The DEGs of PS, BA, and XY in seven modules were clustered by weighted gene co-expression network analysis (WGCNA) (Supplementary Figure 6 and Supplementary Table 13). The genes in brown and black modules showed elevated expression within the PS domain of *PagDET2*-OE lines. GO enrichment analysis indicated that enriched genes were involved in glucose metabolism, nitrogen metabolism, chromatin modification, small peptide synthesis, photosynthesis, cell division, and vascular tissue development. They can also participate in some ethylene response pathways. The enriched genes from turquoise, green, and red modules showed elevated expression within the BA domain of *PagDET2*-OE lines. The GO enrichment showed that these genes were mainly responsible for auxin transport, small peptide synthesis, programmed cell death, secondary cell wall synthesis, and vascular meristem growth (Supplementary Figure 7 and Supplementary Table 14).

## Discussion

The wood biomass accumulation in woody trees is determined by the cambium meristem cell division and XY cell differentiation. Overexpression of *PtoDWF4, PtCYP85A3, PtoDET2*, *and PagDET2* in poplar can increase the content of endogenous BRs, enhancing the wood formation (Jin et al., 2017; Shen et al., 2018; Du et al., 2020; Fan et al., 2020). However, the molecular mechanism of BRs in vascular cambium activity remains to be further elucidated.

In this study, the endogenous CS level was elevated in the poplar stem through overexpressing *PagDET2*, which encodes a 5α-reductase and acts as an early rate-limiting enzyme in the BRs synthesis pathway. *PagDET2* overexpression lines promoted apical and radial growth, thus enhancing stem height and stem diameter in the transgenic trees. The histology analysis showed that the cell numbers of Ph, vascular cambium, and XY domains were significantly increased within the secondary vascular tissues. The *PagDET2* overexpression lines used in this experiment had a better growth phenotype than before used in a previous study (Du et al., 2020), which may be due to the greater increase in gene expression level.

We further analyzed the gene expression changes in response to the elevated endogenous CS signal in shoot apical and secondary vascular stems at the whole genome level. Genes involved in PS cell division and differentiation were significantly enriched in the DEGs of the PS domain. Genes involved in cambium meristem cell division and differentiation, vascular tissue development, and secondary cell wall synthesis related to cellulose and lignin metabolism were significantly enriched in the DEGs of the BA and XY domains. Representative gene, such as *CYCB1; 4*, related to the cambium cell division, was significantly elevated in the BA domain of *PagDET2* overexpression lines. XTHs are involved in polysaccharide metabolism and play an important role in the assembly, decomposition, and remodeling of the cellulose/xylan network (Eklof and Brumer, 2010). *XTH5* and *XTH33* were up-regulated in the BA and secondary XY domain in the *PagDET2* overexpression lines.

Brassinosteroids can collaborate with GA and ethylene signal pathways in regulating cell division and differentiation (Gallego-Bartolome et al., 2012; Vahala et al., 2013; Unterholzner et al., 2015; Zhao et al., 2021). *GA20ox2* is elevated in the PS of *PagDET2* overexpression lines. Overexpression of *GA20ox* in hybrid poplar can enhance XY differentiation and increase the length of wood fibers (Eriksson et al., 2000; Mauriat and Moritz, 2009). *ACO1* was elevated in the XY domain of *PagDET2* overexpression lines. Overexpression of *PttACO1* can stimulate the division activity of cambium and promote the growth of XY (Andersson-Gunneras et al., 2003; Love et al., 2009). The result indicates that GA, ethylene signal pathways, and BRs synergistically regulate vascular tissue development in woody plants, which provides good inspiration for our subsequent research. In summary, the elevated CS level by *PagDET2* can enhance the shoot elongation and stem internode numbers through elevating cell division and GA-related gene expression in the primary growth stem and enhance the stem radial growth and XY differentiation by elevating the ethylene response and secondary cell wall-related genes in the secondary growth stem. A working model of *PagDET2* in regulating woody poplar tree growth is shown in Supplementary Figure 8.

No significant difference was found in the growth between the artificial microRNA material (amiR-*DET2*) and wild-type controls. The synthesis of BR is a multiple enzyme involved complex process. The non-significant difference in *PagDET2* knockdown lines may be due to the compensation mechanisms from other enzymes in the pathway.

The analyses of the comprehensive secondary vascular tissue cell development and transcriptome data in this study provide a useful genomic resource for the woody tree research community. This study also provides molecular insights into the transcriptomic network underpinning vascular cambium and wood formation in poplar.

## Data availability statement

The data presented in this study are deposited in the GEO repository, accession number GSE176470.

## Author contributions

YW, YG, and JD performed the phenotype analysis of *PagDET2* transgenic lines. YW, YH, and JD carried out the RNA-seq data analysis. JD and HS designed the experiments. YW and JD wrote the manuscript. All authors contributed to the article and approved the submitted version.

## Footnotes

1
https://phytozome-next.jgi.doe.gov

2
http://p-sams.carringtonlab.org/

3
http://www.metware.cn/

## Supplementary material

Supplementary Figure 1 Multiple alignments of DET2 amino acid sequences in *Arabidopsis thaliana (At)*, *Gossypium hirsutum (Gh)*, *Populus trichocarpa (Ptr)*, and *Populus alba* × *Populus glandulosa (Pag)*.

Supplementary Figure 2 The expression trend of *DET2* in vascular tissues of *Populus trichocarpa*.

Supplementary Figure 3 The phenotype of *PagDET2* knockdown lines and wild-type at 60-day-old.

Supplementary Figure 4 The anatomy analysis of wild-type and *PagDET2* overexpression lines. **(A)** Toluidine blue stained cross-sectional views of stem between different internodes (IN3, 5, 7, 9) of 45-day-old wild-type and *PagDET2* overexpression lines. **(B–D)** The cross-sectional diameter, Ph thickness, and XY thickness of wild-type and *PagDET2* overexpression lines. IN, internode; XY, xylem; Ph, phloem. Mean ± SD. *Indicate significant difference at *P* < 0.05 (Student’s *t*-test). Bars were indicated on the figures.

Supplementary Figure 5 The shoot apical meristem, the first, second, and third elongating stem internodes were collected and pooled together as the PS. The secondary stem internodes from 5th to 12th were cut, and the bark tissues, which include differentiating phloem and cambium meristem cells, were harvested and named BA. The surface tissues of the trunk were harvested as XY. PS, primary stem; BA, bark; XY, xylem.

Supplementary Figure 6 Gene co-expression analysis. The dendrogram showed the expression trends of differential genes in different tissues between *PagDET2* overexpression lines and wild-type controls, and the dendrogram was divided into seven different modules according to the similarity of expression trends.

Supplementary Figure 7 Gene ontology (GO) functional enrichment analysis on different gene expression modules. Scale is shown above the plot.

Supplementary Figure 8 Working model of *PagDET2* in regulating wood formation in poplar.

Supplementary Table 1 Sequence of the DET2 amino acid.

Supplementary Table 2 Sequence of the primers used in this study.

Supplementary Table 3 Growth comparison of *PagDET2* overexpression and wild-type controls.

Supplementary Table 4 Differentially expressed genes (DEGs) identified by comparing DET2-OE-PS with 84K-PS.

Supplementary Table 5 Differentially expressed genes (DEGs) identified by comparing DET2-OE-BA with 84K-BA.

Supplementary Table 6 Differentially expressed genes (DEGs) identified by comparing DET2-OE-XY with 84K-XY.

Supplementary Table 7 Gene ontology (GO) enrichment analysis of the differentially expressed genes (DEGs) in the PS domain.

Supplementary Table 8 Gene ontology (GO) enrichment analysis of the differentially expressed genes (DEGs) in the BA domain.

Supplementary Table 9 Gene ontology (GO) enrichment analysis of the differentially expressed genes (DEGs) in the XY domain.

Supplementary Table 10 KEGG enrichment analysis of the differentially expressed genes (DEGs) in the primary stem (PS) domain.

Supplementary Table 11 KEGG enrichment analysis of the differentially expressed genes (DEGs) in the bark (BA) domain.

Supplementary Table 12 KEGG enrichment analysis of the differentially expressed genes (DEGs) in the xylem (XY) domain.

Supplementary Table 13 Gene annotation of each module.

Supplementary Table 14 Gene ontology (GO) enrichment analysis of each module genes.
