# Supplementary figures and images for: PagDET2 promotes cambium cell division and xylem differentiation in poplar stem
Source: Front Plant Sci. 2022 Aug 26;13:923530. doi: 10.3389/fpls.2022.923530 (PMC9459238; doi:10.3389/fpls.2022.923530)

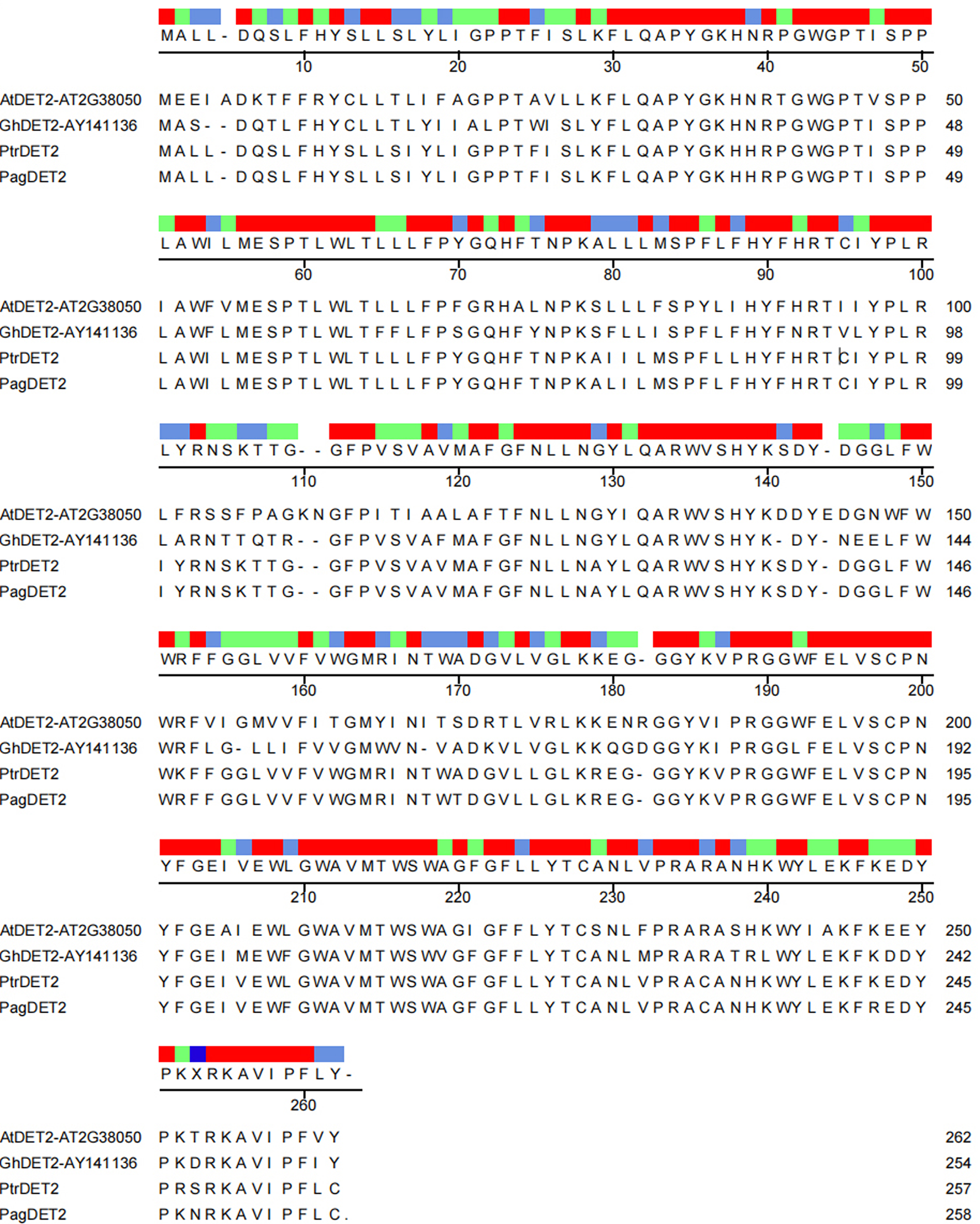

Supplement: Supplementary Figure 1 — Multiple alignments of DET2 amino acid sequences in Arabidopsis thaliana (At), Gossypium hirsutum (Gh), Populus trichocarpa (Ptr), and Populus alba × Populus glandulosa (Pag). [file Image_1.TIF]

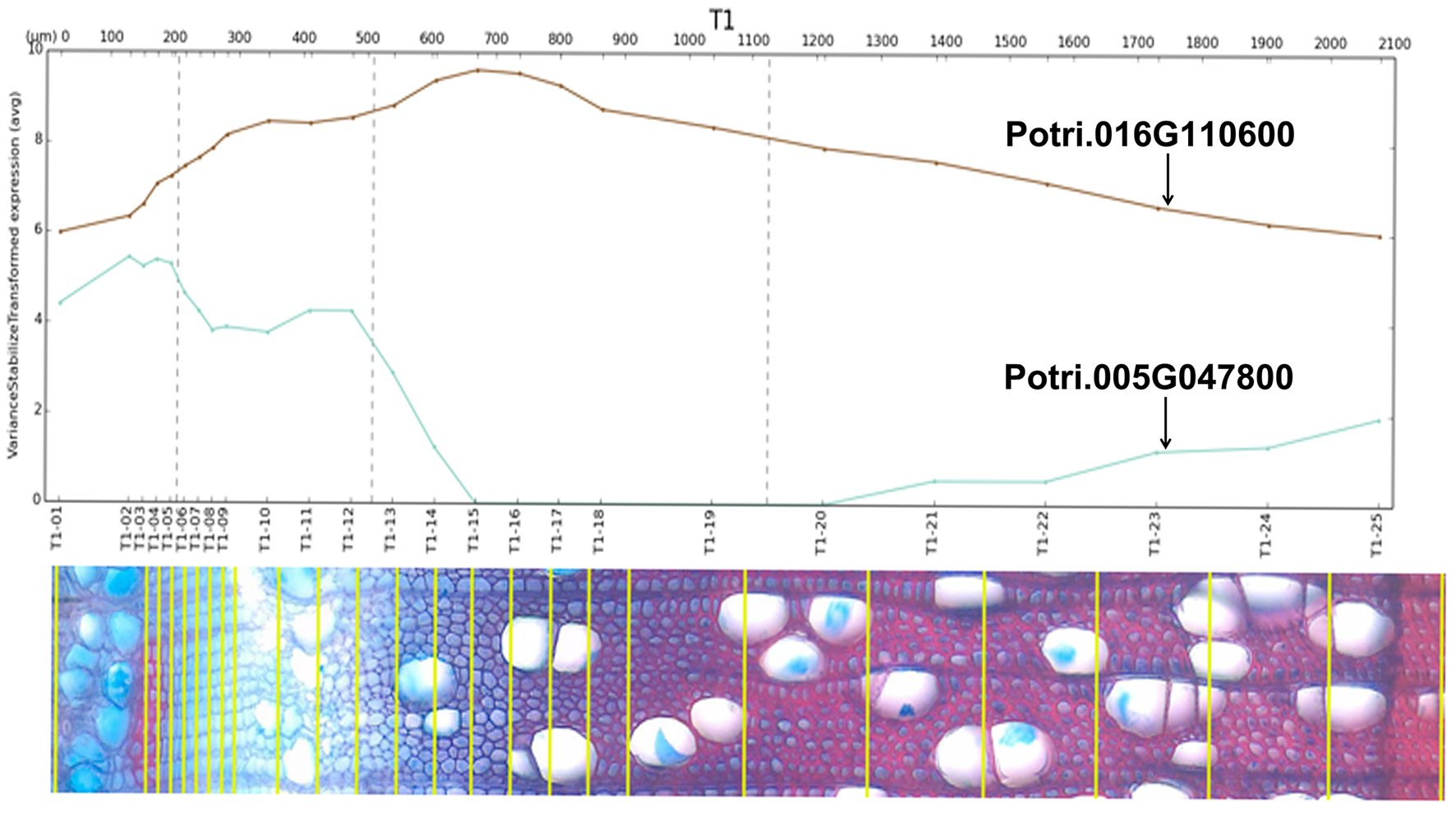

Supplement: Supplementary Figure 2 — The expression trend of DET2 in vascular tissues of Populus trichocarpa. [file Image_2.TIF]

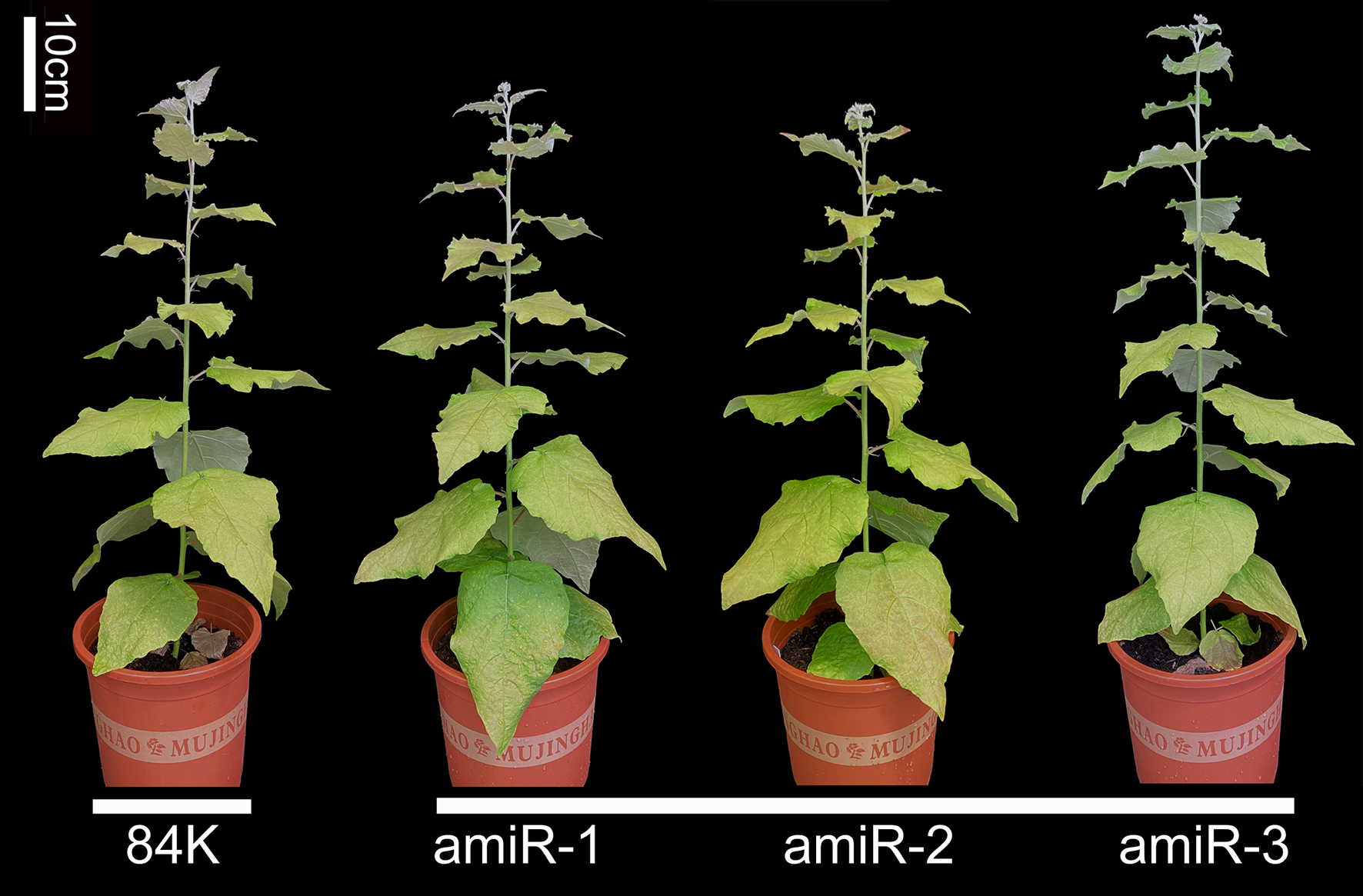

Supplement: Supplementary Figure 3 — The phenotype of PagDET2 knockdown lines and wild-type at 60-day-old. [file Image_3.TIF]

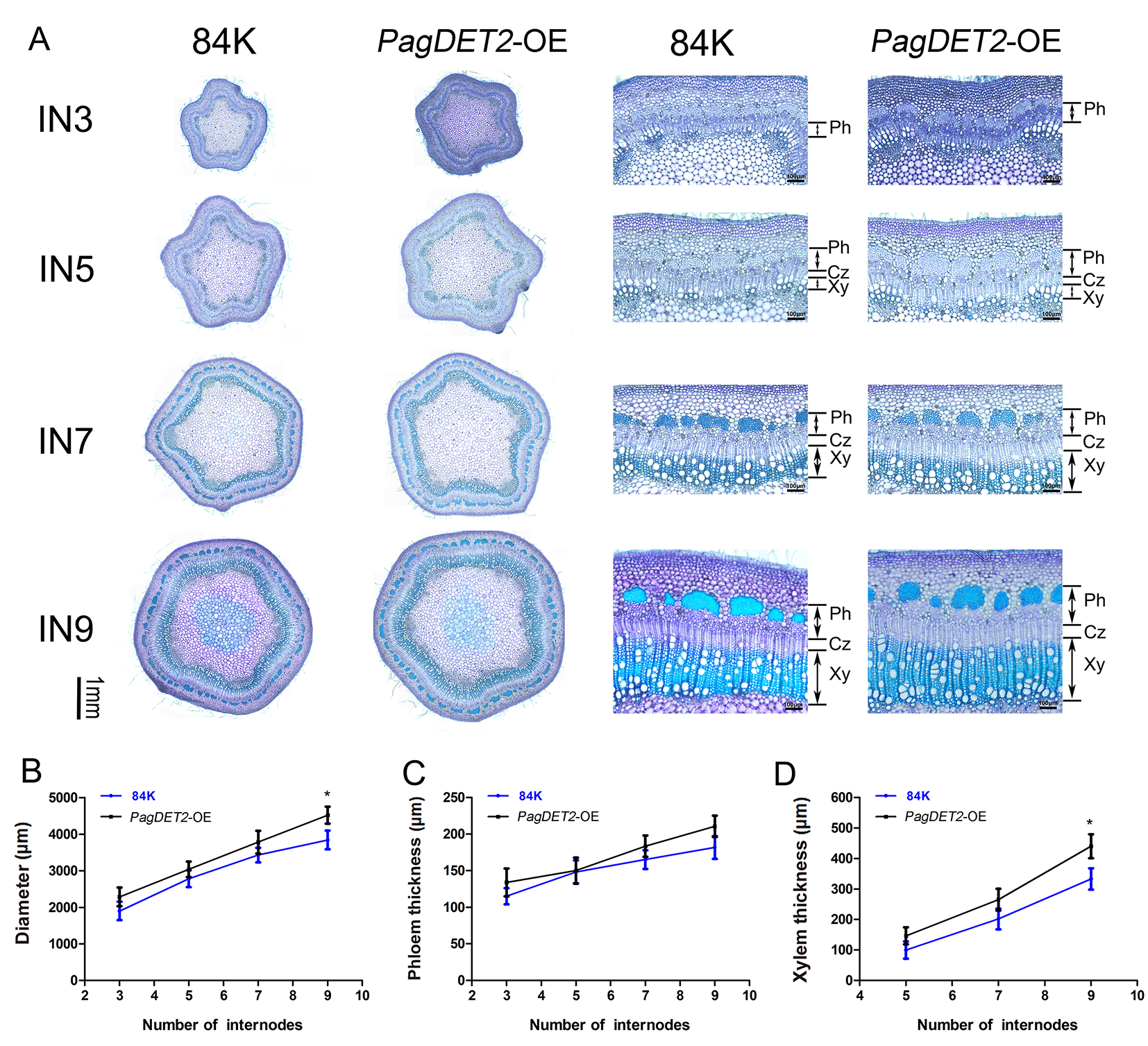

Supplement: Supplementary Figure 4 — The anatomy analysis of wild-type and PagDET2 overexpression lines. (A) Toluidine blue stained cross-sectional views of stem between different internodes (IN3, 5, 7, 9) of 45-day-old wild-type and PagDET2 overexpression lines. (B–D) The cross-sectional diameter, Ph thickness, and XY thickness of wild-type and PagDET2 overexpression lines. IN, internode; XY, xylem; Ph, phloem. Mean ± SD. *Indicate significant difference at P < 0.05 (Student’s t-test). Bars were indicated on the figures. [file Image_4.TIF]

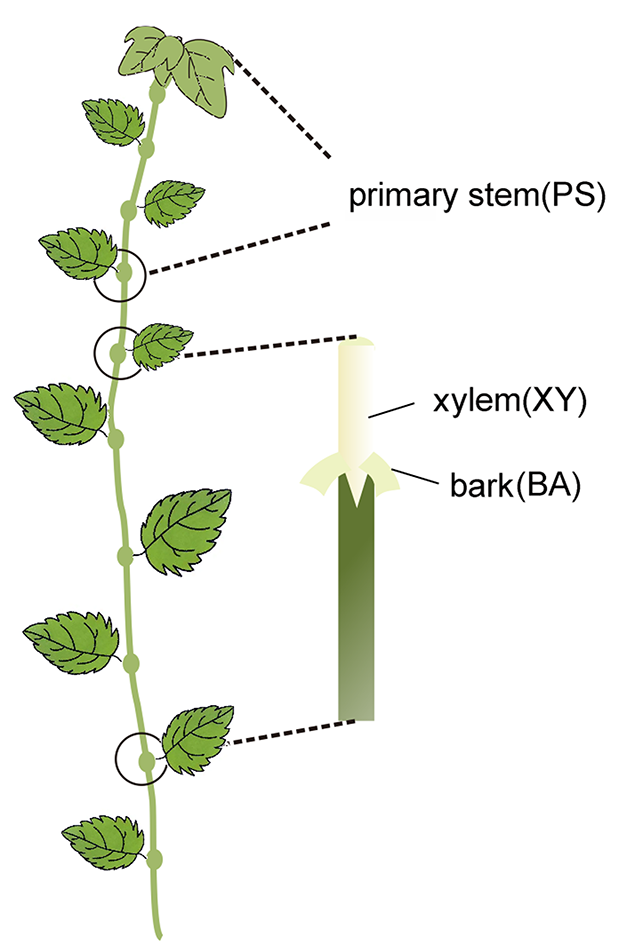

Supplement: Supplementary Figure 5 — The shoot apical meristem, the first, second, and third elongating stem internodes were collected and pooled together as the PS. The secondary stem internodes from 5th to 12th were cut, and the bark tissues, which include differentiating phloem and cambium meristem cells, were harvested and named BA. The surface tissues of the trunk were harvested as XY. PS, primary stem; BA, bark; XY, xylem. [file Image_5.tif]

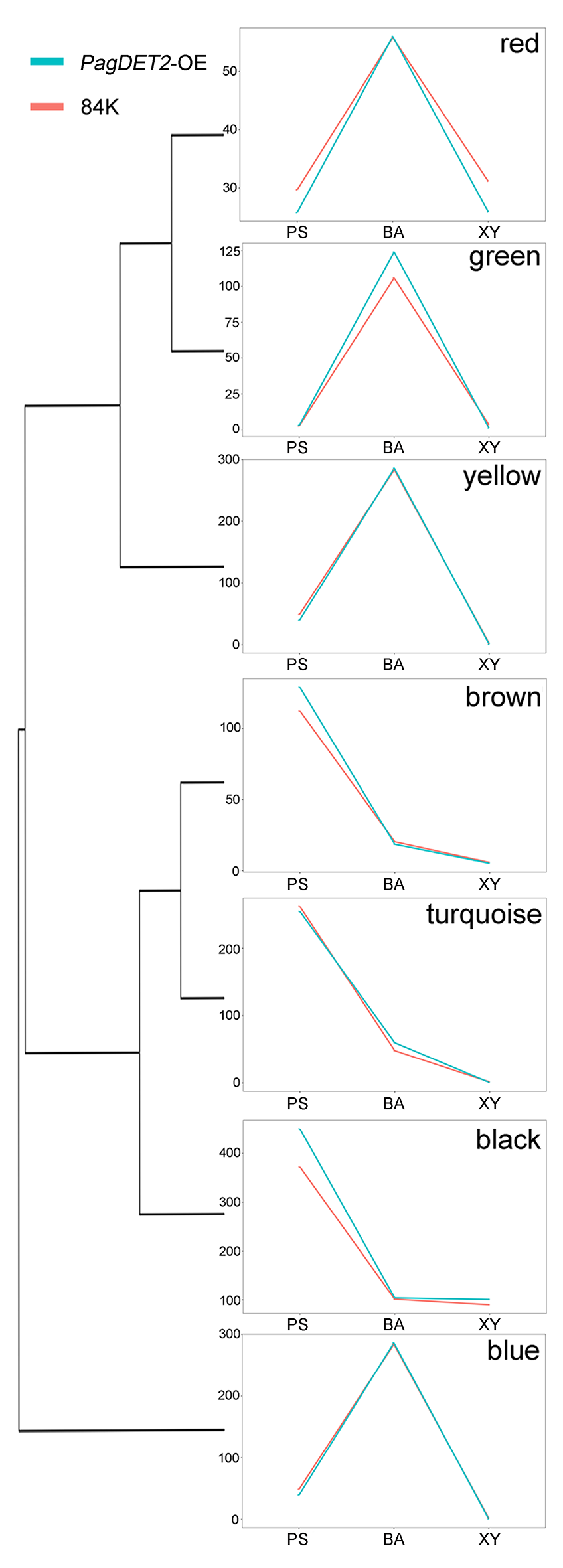

Supplement: Supplementary Figure 6 — Gene co-expression analysis. The dendrogram showed the expression trends of differential genes in different tissues between PagDET2 overexpression lines and wild-type controls, and the dendrogram was divided into seven different modules according to the similarity of expression trends. [file Image_6.TIF]

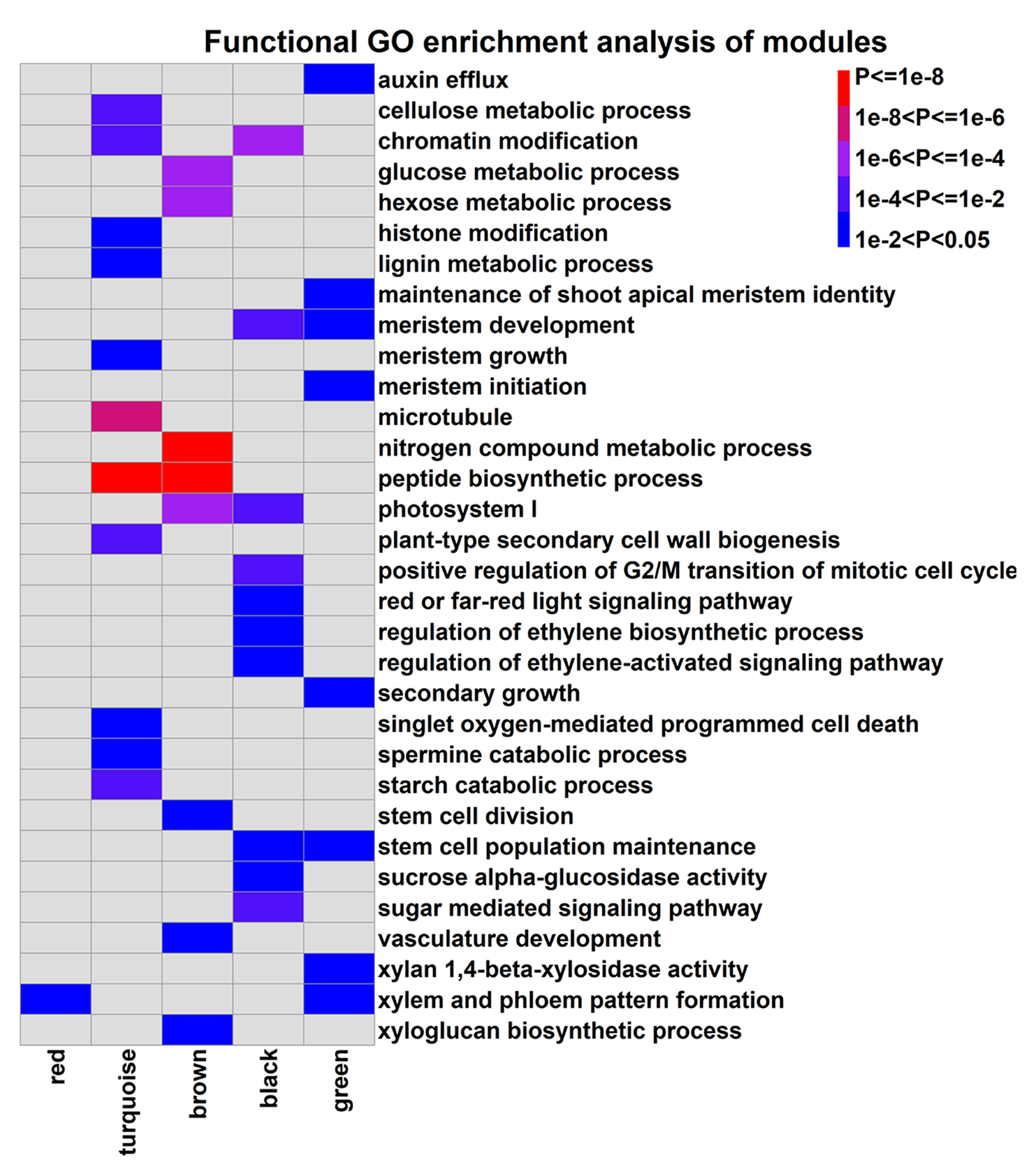

Supplement: Supplementary Figure 7 — Gene ontology (GO) functional enrichment analysis on different gene expression modules. Scale is shown above the plot. [file Image_7.TIF]

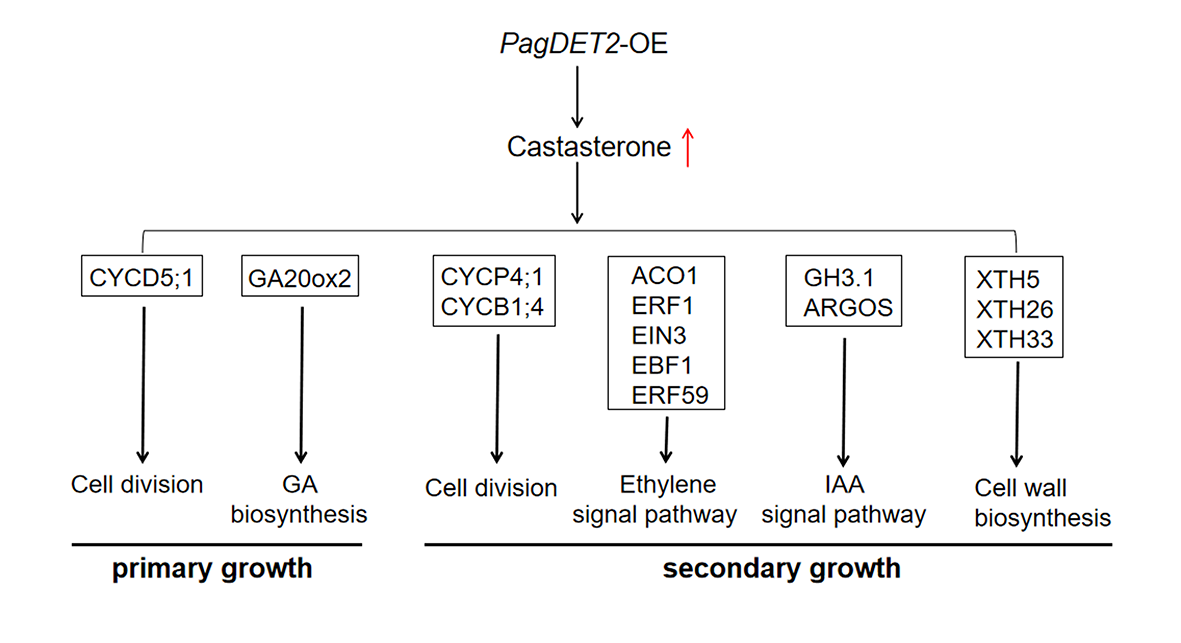

Supplement: Supplementary Figure 8 — Working model of PagDET2 in regulating wood formation in poplar. [file Image_8.tif]
